# Participant Recruitment and Retention in Remote eHealth Intervention Trials: Methods and Lessons Learned From a Large Randomized Controlled Trial of Two Web-Based Smoking Interventions

**DOI:** 10.2196/10351

**Published:** 2018-08-24

**Authors:** Noreen L Watson, Kristin E Mull, Jaimee L Heffner, Jennifer B McClure, Jonathan B Bricker

**Affiliations:** ^1^ Fred Hutchinson Cancer Research Center Seattle, WA United States; ^2^ Kaiser Permanente Washington Health Research Institute Seattle, WA United States; ^3^ University of Washington Seattle, WA United States

**Keywords:** recruitment, retention, randomized controlled trial, RCT, smoking cessation, web intervention

## Abstract

**Background:**

Despite having many advantages, online eHealth trials are not without challenges—notably, participant recruitment, and outcome data retention. Moreover, publications from these trials rarely provide detailed information on the methods used for recruitment and retention or discuss implications of the methods for future studies.

**Objective:**

To address this need for empirical guidance regarding recruitment and outcome data retention planning, we aim to describe the methods and lessons learned from the recruitment and retention procedures used in a large randomized trial of 2 Web-based smoking cessation interventions.

**Methods:**

To ensure a demographically and geographically diverse participant sample, we used the recruitment strategies (1) traditional, (2) Web-based, and (3) online survey panel methods and adaptively modified each in response to recruitment success. At baseline, participants indicated how they heard about the study and answered demographic questions. To maximize trial retention at each of the 3-, 6-, and 12-month assessment points, 4 survey modalities (first Web, followed by phone, mail, and postcard) were sequentially timed over a 30-day period. Participants received US $25 for submitting their responses, regardless of modality, and received an additional US $10 bonus for completing the Web survey within 24h of electronic notification.

**Results:**

We randomized 2637 smokers in 16 months and achieved 88% retention at 12-months. Participants (79.26% female, 72.60% Caucasian) were recruited from all 50 states. The majority of participants were recruited through Facebook (49.43%), followed by the survey panel (20.85%), free internet sources (14.54%), traditional media (11.34%), and Google ads (3.84%). Descriptively, participant demographics varied by recruitment source. Of the completed follow-up surveys, most were completed by Web (92%). Retention rates did not vary by recruitment source.

**Conclusions:**

Continuous monitoring and refinement of multiple recruitment methods, particularly of online advertising campaigns, allowed us to maximize the effectiveness of recruitment strategies in recruiting a large, diverse sample of smokers. Likewise, offering multiple follow-up survey modalities in sequential order along with time-dependent bonus incentives enabled us to obtain outcome data from a very high level of enrolled participants for the duration of the trial protocol. These strategies may be similarly useful in other trials.

**Trial Registration:**

ClinicalTrials.gov NCT01812278; https://clinicaltrials.gov/ct2/show/NCT01812278 (Archived by WebCite at http://www.webcitation.org/71gy5GLvO)

## Introduction

In recent years, access to the internet has climbed exponentially worldwide [1]. In parallel with this global trend, more eHealth interventions are being developed and tested in online trials. In fact, as of June 2018, searching “eHealth” in PubMed yields over 29,000 results. Considering the potential for high reach and opportunity to provide low-cost interventions, one of the prominent allures of eHealth interventions is the possibility for them to make population-level health impacts. Moreover, conducting eHealth intervention research online offers a multitude of advantages including automated data collection, high control over intervention content and format, low cost, maximizing external validity, and potential for rapid recruitment of large numbers of participants [2,3]. However, online eHealth trials are not without challenges—most notably, recruitment of research participants and outcome data retention.

Although not unique to online trials [4,5], many online eHealth studies have difficulty obtaining adequate sample size [2,6-9] and recruiting a representative sample of their target population [10-12]. Such difficulties with participant recruitment can result in extended recruitment time, increased costs for recruitment, as well as inadequate statistical power if accrual targets are not met [13]. In fact, failure to meet accrual targets is the primary reason for premature trial termination [4,14,15].

Another major methodological challenge faced by online eHealth trials is keeping participants engaged in the study protocol and achieving adequate rates of outcome data retention [2,9,16-19]. Low retention rates increase the risk of selection bias (particularly if there are differential retention rates between arms), threaten validity, and lead to loss of statistical power [5,17,20]. Moreover, using tobacco treatment studies as an example, methods commonly used to address issues of low retention (eg, imputing all missing outcome data as smoking) are also problematic in that they can lead to inflated type-I and type-II errors [21]. For these reasons, drawing conclusions from studies with low retention rates, even when using conventional methods of imputation [21], can be misleading.

At present, best-practice standards do not exist for how to recruit or retain participants in remotely conducted eHealth intervention trials and most trials do not detail their recruitment and retention methods sufficiently so that others can learn from their successes or mistakes. Among the remotely conducted trials of eHealth interventions that report a more significant amount of detail, there is considerable variability in the amount and type of information provided on the trial recruitment and retention strategies [3,22-26]. As a result, much of the information is not generalizable, it is difficult to determine which methods are most effective under what circumstances, and little is known about how recruitment sources might affect participant characteristics or data retention. Greater transparency of these issues has, therefore, been strongly encouraged in the existing literature [6,11,14,27,28] as detailed reports could inform plans for recruitment and retention in future trials of eHealth interventions.

Thus, the primary aim of this paper is to provide a detailed description of the recruitment and outcome data retention methods used in a successful online eHealth trial (WebQuit), including lessons learned that might be useful for future eHealth trials. The WebQuit trial compared the effectiveness of 2 online smoking cessation programs in a diverse sample of smokers (N=2637) recruited across the US; 88% of participants completed the one-year follow-up survey [29]. To inform future strategies, we also examine the effects of recruitment source on participant characteristics and outcome data retention as well as the effects of participant characteristics on data retention.

## Methods

### Overview of WebQuit Trial Design

The WebQuit trial was conducted to compare the effectiveness of 2 Web-based interventions for smoking cessation among adult smokers [29]. The websites evaluated in the trial were (1) a Web intervention based on Acceptance and Commitment Therapy (WebQuit.org), and (2) the National Cancer Institute’s Smokefree.gov website, which is the most accessed cessation website worldwide. The interventions were designed to be stand-alone interventions. Thus, the trial involved minimal contact with study personnel and did not provide pharmacotherapy to study participants. In addition, to access to their randomly assigned program, participants in both arms could receive up to four messages per day (via text or email) designed to encourage engagement with their assigned website, unless they opted out. These messages were sent for the first 28 days after randomization. Participant follow-up data were collected at 3, 6, and 12 months after randomization.

#### Target Population.

Participants (N=2637) were adult smokers living in the US. The eligibility requirements for the study included: (1) ≥ 18 years old, (2) smoke ≥5 cigarettes per day for the last year, (3) desire to quit smoking within 30 days, (4) have access to high-speed internet and email, (5) not participating in other cessation interventions or treatment, (6) never having used Smokefree.gov, (7) never having participated in one of our previous studies, (8) have no other household member participating in the study, (9) willingness to be randomized to treatment, complete 3 outcome surveys, and to provide contact information for themselves and 2 relatives, (10) live in the US, and (11) the ability to read in English. To recruit a diverse sample with adequate representation of smokers identifying as racial and ethnic minorities, we aimed to recruit a sample comprised of at least 25% smokers identifying as a racial/ethnic minority (ie, smokers who do not identify as non-Hispanic Caucasian). The target sample size for the study, which was based on having 80% power to detect a two-tailed significant difference between quit rates estimated for the 2 arms from our pilot study and relapse rates [29], was met.

### Recruitment Strategy

We recruited participants for 18 months, from March 2014 to August 2015. We implemented a multi-pronged recruitment strategy that encompassed traditional, Web-based, and online survey panel methods (detailed below). Recruitment methods were monitored on an ongoing basis and modified as needed based on recruitment success. Our recruitment strategy was based on methods used in the pilot trial of WebQuit [23,30]. Also, we consulted with the Dana-Farber/Harvard Cancer Center Health Communication Core (DF/HCC) to develop advertisements, a recruitment website, and logo that would be relevant, sensitive, and appealing to our target population and distinguishable from other tobacco cessation websites. The DF/HCC also provided ongoing consultation regarding online advertising strategies for Facebook and Google. To ensure that our data retention operations team would have the capacity to handle the procedures for collecting follow-up data (discussed below), we limited the number of participants able to enroll in the study per month. We were able to closely monitor the study flow and ensure data quality by putting restraints on how quickly we recruited participants. All sources of recruitment directed interested individuals to a study website. The purpose of the website was to establish the credibility of the study and communicate the purpose of the study, how to enroll, and what would be asked of participants. Individuals who consented to be screened for the study were directed to a short screening survey to determine basic eligibility criteria. Individuals deemed eligible by the screening survey were immediately sent an email directing them to an online informed consent.

#### Traditional Recruitment Methods

Traditional methods for recruitment included press releases as well as newspaper, radio, and television interviews with the principal investigator (JBB). Participants recruited through targeted mailed invitations to known smokers within a large regional health plan (approximately 10,000 letters mailed in batches from December 2014 to August 2015), hospitals, or word of mouth by friends, family, or health care providers were also considered as being recruited through traditional methods.

#### Free Internet Methods

Participants were recruited through several free Web-based sources including Craigslist, Reddit, visibility of our recruitment website on search engines, and information about the study seen or shared on local and national websites (eg, news websites, fredhutch.org, the Penny Hoarder, Twitter). For recruitment on Craigslist, 296 advertisements were made throughout the recruitment period, with most posts made during the first 10 months. Two advertisements were made per day until all predetermined areas across 18 states were posted in. Predetermined areas were selected based on smoking prevalence [31], economic status, and rural areas (Alaska, Arizona, Arkansas, Indiana, Kentucky, Louisiana, Michigan, Mississippi, Missouri, Nevada, North Dakota, Ohio, Oklahoma, South Carolina, South Dakota, Tennessee, Virginia, and West Virginia). Additional postings were made in 18 cities with high concentrations of smokers [32] (eg, Wilkes Barre-Scranton, PA; Birmingham, AL; Grand Rapids, MI) and in 24 cities with high percentages of Black and African American populations (eg, Miami Gardens, FL; Birmingham, AL; Baltimore, MD). Some areas received up to three posts over the recruitment period.

**Figure 1:**
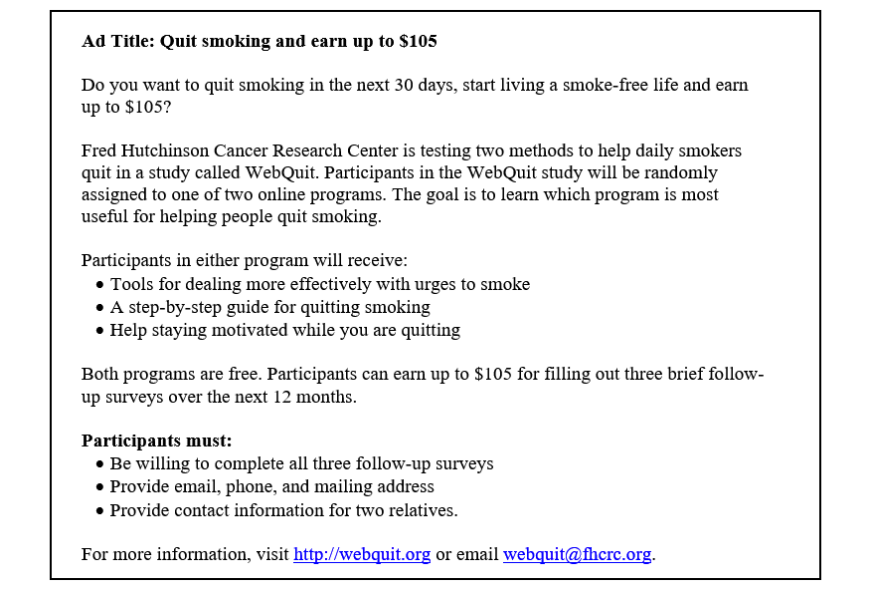
Content used for general Craigslist advertisements.

In addition to a general study advertisement, a season-specific advertisement was placed in several cities around January 2015. Ads were placed in the “Community” tab under both the “General Community” and “Volunteers” subsections of all cities. An example of the wording used in the Craigslist ads can be found in Figure 1. For recruitment on Reddit, 9 posts with similar wording were made throughout the recruitment period in different Reddit subgroups (eg, SampleSize, stopsmoking, addiction).

#### Facebook Advertisements

**Figure 2:**
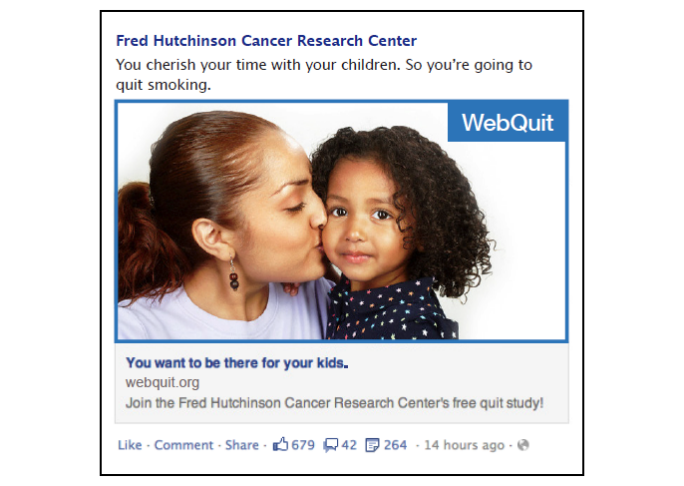
Example Facebook ad with image.

In consultation with the DF/HCC, 17 Facebook ads were created that varied in the images and wording used (see Figure 2 for an example ad) to be relevant to our target population. Most images were of cigarettes, the Fred Hutchinson Cancer Research Center logo, or people. Images of people varied by gender, race/ethnicity, the age of the person/people in the photo, and in how many people were pictured (1 to 3). Wording varied around eight content categories: (1) health (“You heard your doctor. And you’re ready to quit smoking.”), (2) readiness to quit (“You’re ready to quit smoking and we’re ready to help!”), (3) relationships (“You cherish your time with your children. So you’re going to quit smoking.”), (4) freedom from cigarettes (“You’re ready to be free, You’re ready to quit smoking.”), (5) research (“Earn up to $105 to quit with us—free, online quit smoking study from the Fred Hutchinson Cancer Research Center!”), (6) financial (“You’re ready to spend money on what YOU want, not on cigarettes!”), (7) appearance (“You’re ready to look better, smell better—BE better! Are you ready to quit smoking?”), and (8) help/altruism (“You’re ready to quit smoking. We’re ready to share free skills and support to help you quit!”).

Creating multiple ads allowed us to determine which were most effective in real time. For the first 16 days of the ad campaign, advertisements were run in two-day intervals to determine which permutations of images and wording were most successful.

Afterward, the top 3 ads were run one at a time so that Facebook’s embedded algorithm could optimize ad performance. One of the most successful ads read, “You heard your doctor. And you’re ready to quit smoking. We have a great opportunity—earn up to $105!” Ads were turned on and off in response to which were yielding the highest rates of randomization into the trial at the lowest cost per randomization. To increase the likelihood of reaching our desired population, we set several targeting parameters including ages 18-65 or older, English speaking, US, and people who identified relevant interests (eg, cigarette, quit smoking, electronic cigarette). Facebook ads ran from March 2014 until April 2015. Per recommendations from the DF/HCC and Facebook, we continued to monitor the performance of the ad campaign as response drop-offs are common over time. Minor adjustments were made as needed to boost ad performance.

#### Google Advertisements

Google ads were created with the same wording as the Facebook ads. Twenty keywords determined by the study team and DF/HCC that were associated with the study theme (eg, “quit smoking,” “help to quit smoking,” “stop smoking,” “how do I stop smoking,” “tips to quit smoking”) were rotated through to optimize response. Similar to the strategy used for Craigslist postings, we set parameters to target areas based on high prevalence of smoking, economic status, and rural areas. However, in monitoring conversions from Google ads, it quickly became apparent that the ads were not performing as well as Facebook ads (ie, lower rates of randomization). As a result, and to maximize recruitment efforts and resources, we discontinued the Google ads after approximately two months (March-May 2014).

#### Online Survey Panel

To help boost recruitment of minority smokers, we utilized the online survey panel company Survey Sampling International (SSI) [33] beginning in April 2015. Such companies can send study announcements to members of their online panels who meet specific criteria. For this study, we requested that they recruit racial/ethnic minority smokers living in the US. The panels are comprised of verified individuals who have voluntarily signed up to participate in online surveys and research in exchange for incentives. SSI was able to identify members of their panel who were likely to be eligible for the study and targeted the audience based on behavioral and demographic characteristics. Respondents recruited from the survey panel answered a brief screener to ensure they were the target audience before being directed to the study website.

In addition to utilizing SSI to boost enrollment of minority smokers, we also limited enrollment on non-minority smokers beginning in February 2015 because we would otherwise not have reached our goal of enrolling at least 25% minority smokers. Specifically, the screening survey was programmed with an algorithm that would randomly reject a set proportion of non-Hispanic Caucasian smokers that were otherwise eligible. For example, if the rejection rate was 30%, non-minority smokers were randomly assigned a number between 0 and 1. If that number were less than 0.30, they would be ineligible. To ensure we met our recruitment goal, the rate of rejection varied over time.

All activities for participant recruitment were coordinated by our project manager in consultation with the study team. On average, the project manager spent an estimated hour per day on recruitment activities throughout the recruitment period, with more time spent at the beginning of the recruitment period. Primary recruitment-related activities included communicating with outside vendors (DF/HCC, SSI, media) and setting up contracts as necessary, reviewing enrollment reports and communicating the enrollment status to key stakeholders, making daily posts to Craigslist (as discussed above), monitoring and tweaking Google and Facebook ads, and communications with our data retention operations team.

### Enrollment

All enrollment procedures occurred online by way of the study website noted above. Interested individuals completed a screening survey to assess eligibility criteria. Antifraud measures were also implemented to decrease the likelihood of fraudulent participation (eg, enrolling more than once, changing survey responses to become eligible). Specifically, these measures included reviewing internet protocol addresses for duplicates or non-US origin, CAPTCHA authentication, and review of survey logs for suspicious response times (ie, completing the screening assessment in <90 seconds or completing the baseline survey in <10 minutes). Study staff contacted individuals with suspicious responses to confirm their information. If their information could not be confirmed, they were not enrolled in the study [29]. Eligible individuals were sent an e-mail from the study inviting them to return to the study website to provide informed consent, complete a baseline survey, and provide contact information. Eligible individuals had 14 days to complete the online enrollment process. Automated reminder emails were sent to eligible individuals on days 5 and 11 if they had not returned to the website. On day 7, a personalized email from study staff was sent in case the emails from the study email address were sent to participants’ spam folder. After completing the baseline assessment, participants had 30 days to return to the study website for randomization. Up to three weekly email reminders were sent from the study email address to participants who had not returned to the website. Ultimately, 2637 participants were randomized into the trial.

### Measures

In the initial screening survey, participants reported how they learned about the study by selecting from one of 13 response options, including an “other” category in which they were able to write out an answer. For this manuscript, response options were grouped into the 5 recruitment methods described above: (1) traditional, (2) free-internet, (3) Facebook ads, (4) Google ads, and (5) online survey panel. We were able to classify all but 9 participants into 1 of the recruitment categories. While we were able to confirm participants recruited through the survey panel, all other responses are self-reported, which is typical for research on recruitment methods [22,34,35]. The baseline assessment also included questions about demographic characteristics and validated self-report screening measures of the following mental health conditions: depression (Center for Epidemiologic Studies Depression scale) [36], generalized anxiety (Generalized Anxiety Disorder 7-item scale) [37], panic disorder (Autonomic Nervous System Questionnaire) [38], posttraumatic stress disorder (PTSD; PTSD Checklist) [39], and social anxiety disorder (Mini-Social Phobia Inventory) [40]. A geographic classification of participants was determined by linking the participants’ zip codes to Rural-Urban Commuting Area (RUCA) codes [41]. There are 10 primary classifications based on population density, urbanization, and daily commuting. Definitions of the 10 RUCA codes can be found on the US Department of Agriculture website [41]. Zip codes associated with RUCA codes 1-6 were classified as metropolitan/micropolitan while those associated with RUCA codes 7-10 were classified as small town/rural areas.

### Retention Strategy

Follow-up data were collected at 3, 6, and 12 months after randomization. To maximize data retention at each follow-up assessment, participants had up to 30 days to complete the assessments. Four survey modalities were sequentially timed until the survey was completed. Sequentially timing survey modalities has been shown to improve response rates compared to offering multiple survey modalities in parallel [42]. The modalities were (1) Web, (2) telephone, (3) mailed survey, and (4) a postcard with selected outcomes.

For each follow-up assessment, we utilized the following strategy until participants completed the survey. Two weeks before the Web-based follow-up survey was available participants were mailed a survey invitation with a US $2 preincentive. Participants were then sent up to three automated emails with a link to the Web version of the survey on days 0 (exactly 3, 6, or 12 months after randomization), 5, and 9. Afterward, participants had the opportunity to complete the survey via phone. Study staff called participants up to eight times, once per day on days 10-17. On day 18, if participants had not completed the survey, study staff mailed a paper version of the survey with a prestamped and addressed return envelope. If participants did not respond to any of the previous modalities by day 30, they were mailed a postcard that only inquired about primary outcomes and a few selected secondary outcomes.

We incentivized participants with US $25 for completing a survey, regardless of modality. Additionally, to encourage timely responses, participants who completed the Web-based survey within 24 hours of any email received a US $10 bonus. Thus, participants received up to US $105 in incentives for completing follow-up surveys.

### Analyses

Descriptives regarding participant demographics across recruitment sources are reported. To examine the association between participant characteristics and recruitment source and 12-month data retention, we used logistic regression models with a covariate for treatment arm and accounted for multiple comparisons by adjusting *P* values to control the false discovery rate [43]. To assess differences in data retention across recruitment sources, we conducted chi-square tests for total response rates at the 3-, 6-, and 12-month follow-up assessments.

## Results

### Participants

We recruited and randomized 2637 smokers. Of these, 2628 could be classified into 1 of the 5 recruitment categories and were thus retained for these analyses. The mean age of the sample at baseline was 46.15 (SD 13.36) years, and most of the sample was female (2083/2628, 79.26%). A large proportion of participants identified as Caucasian (1908, 72.60%), 278 (10.58%) identified as Black or African American, and 442 (16.82%) identified as another race (Asian, Native American, Native Hawaiian, or more than one race). A total of 222 (8.44%) identified their ethnicity as Hispanic. The remaining demographic characteristics of the overall sample can be found in Table 1.

### Recruitment and Demographic Variation by Recruitment Source

**Figure 3:**
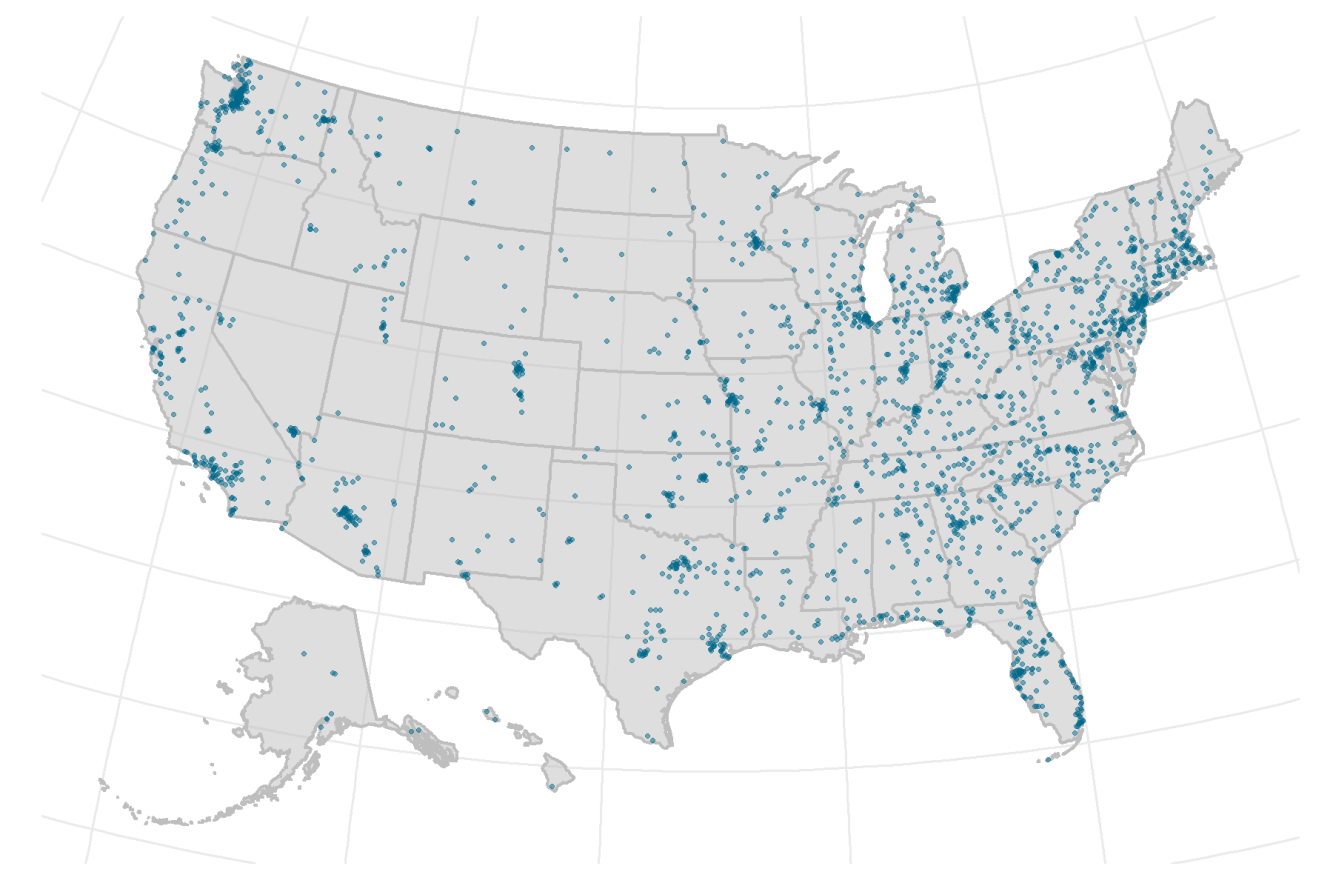
Geographic location of participants from all 50 states; each participant is represented by a single dot.

Most of the sample was recruited from Facebook (1299/2628, 49.43%), followed by the survey panel (548, 20.85%), free internet sources (382, 14.54%), traditional methods (298, 11.35%), and Google ads (101, 3.84%). Using these recruitment channels, we recruited participants from all 50 states (Figure 3).

Most demographic characteristics of the participants varied across recruitment sources (Table 1). Facebook advertisements recruited the oldest smokers with a mean of 52.87 (SD 10.54) years of age, while the survey panel recruited the youngest with a mean of 35.67 (SD 9.81) years of age. Although most smokers from all recruitment sources were women, traditional recruitment sources yielded the highest percentage of males (85/298, 28.52%), while Facebook ads resulted in the lowest (198/1299, 15.24%). The highest proportion of Black and African American smokers were recruited by both Google ads (18/101, 17.82%) and the online survey panel (98/548, 17.88%). As in the targeted recruitment plan, the online survey panel recruited the highest proportion of smokers identifying as Hispanic ethnicity (111/548, 20.26%) and as a race other than Caucasian or Black/African American (196, 35.77%). Google ads recruited the highest proportion of smokers with a high school education or less (37/101, 36.63%) while traditional recruitment sources recruited the highest proportion of smokers with a bachelor’s degree or more (77/298, 25.84%). Free internet sources recruited the highest percentage of participants who identified as lesbian, gay, or bisexual (56/382, 14.66%); with the lowest percentage recruited from Facebook (84/1229, 6.83%). Regarding income, participants recruited from Google ads were most likely to report an income that is greater than US $20,000 (36/101, 35.64%) while participants recruited from the survey panel were most likely to report their income as higher than US $20,000 (432/548, 78.83%). Although most participants in the study lived in metropolitan or micropolitan areas, Facebook recruited the highest proportion of participants from small towns or rural areas (155/1299, 11.93%), whereas traditional methods recruited the lowest proportion from these areas (15/298, 5.03%). All recruitment sources yielded similar proportions of smokers who screened positive for one or more mental health conditions.

**Table 1:** Demographic characteristics by recruitment source.

Parameter		Total (N=2628), n (%)	Recruitment source						*P* value^a^
			Traditional^b^ (n=298), n (%)	Free internet^c^ (n=382), n (%)	Facebook ad^b^ (n=1299), n (%)	Google ad^c^ (n=101), n (%)	Survey panel^d^ (n=548), n (%)			
Age, (years), mean (SD)		46.15 (13.36)	46.29 (12.93)	39.56 (12.97)	52.87 (10.54)	40.96 (14.04)	35.67 (9.81)		<.001
**Age (years)**									<.001
	18-24	145 (5.52)	9 (3.02)	42 (10.99)	24 (1.85)	12 (11.88)	58 (10.58)		
	25-44	984 (37.44)	117 (39.26)	204 (53.40)	229 (17.63)	47 (46.53)	387 (70.62)		
	45-64	1312 (49.92)	148 (49.66)	125 (32.72)	902 (69.44)	38 (37.62)	99 (18.07)		
	>65	187 (7.12)	24 (8.05)	11 (2.88)	144 (11.09)	4 (3.96)	4 (0.73)		
**Gender**									<.001
	Male	545 (20.74)	85 (28.52)	108 (28.27)	198 (15.24)	26 (25.74)	128 (23.36)		
	Female	2083 (79.26)	213 (71.48)	274 (71.73)	1101 (84.76)	75 (74.26)	420 (76.64)		
**Race**									<.001
	Caucasian	1908 (72.60)	229 (76.85)	246 (64.40)	1116 (85.91)	63 (62.38)	254 (46.35)		
	Black/African American	278 (10.58)	22 (7.38)	56 (14.66)	84 (6.47)	18 (17.82)	98 (17.88)		
	Other^e^	442 (16.82)	47 (15.77)	80 (20.94)	99 (7.62)	20 (19.80)	196 (35.77)		
**Ethnicity**									<.001
	Hispanic	222 (8.45)	18 (6.04)	40 (10.47)	43 (3.31)	10 (9.90)	111 (20.26)		
	Non-Hispanic	2406 (91.55)	280 (93.96)	342 (89.53)	1256 (96.69)	91 (90.10)	437 (79.74)		
**Education**									<.001
	≤High school	733 (27.89)	67 (22.48)	99 (25.92)	414 (31.87)	37 (36.63)	116 (21.17)		
	Some college or junior college	1363 (51.86)	154 (51.68)	205 (53.66)	638 (49.11)	45 (44.55)	321 (58.58)		
	≥Bachelor’s degree	532 (20.24)	77 (25.84)	78 (20.42)	247 (19.01)	19 (18.81)	111 (20.26)		
**Sexual orientation**									<.001
	Heterosexual	2375 (90.37)	264 (88.59)	326 (85.34)	1215 (93.53)	91 (90.19)	479 (87.41)		
	LGB^f^	253 (9.63)	34 (11.41)	56 (14.66)	84 (6.47)	10 (9.90)	69 (12.59)		
**Mental health**									.13
	Screened positive for MHC^g^	1930 (73.44)	209 (70.13)	273 (71.47)	933 (71.82)	75 (74.26)	440 (80.29)		
	Did not screen positive	574 (21.84)	72 (24.16)	92 (24.08)	284 (21.86)	23 (22.77)	103 (18.80)		
**Income (US $)**									<.001
	≤20,000	735 (27.97)	83 (27.85)	95 (24.87)	405 (31.18)	36 (35.64)	116 (21.17)		
	>20,000	1892 (71.99)	215 (72.15)	287 (75.13)	893 (68.75)	65 (64.36)	432 (78.83)		
**Location**									<.001
	Metropolitan or micropolitan	2365 (89.99)	280 (93.96)	356 (93.19)	1137 (87.53)	94 (93.07)	498 (90.88)		
	Small town or rural	246 (9.36)	15 (5.03)	24 (6.28)	155 (11.93)	6 (5.94)	46 (8.39)		

^a^Adjusted for false discovery rate.

^b^All advertisements were designed to be culturally sensitive and appealing to our target population; however, no unique population targeting was used for traditional recruitment sources or Facebook ads.

^c^Minimal targeting was used for Craigslist and Google ads in that some locations for posts were chosen to encompass areas with greater smoking prevalence, greater rural areas, and lower economic status. Craigslist ads were also posted in cities with high proportions of Black and African American populations.

^d^The online survey panel was used specifically to boost recruitment of minority (non-Caucasian) smokers.

^e^Asian, Native American, Native Hawaiian, or more than one race.

^f^LGB: lesbian, gay, or bisexual.

^g^MHC: mental health condition.

In assessing baseline participant characteristics associated with data retention (see Table 2), only gender emerged as a significant predictor, with males being less likely to complete the 12-month follow-up assessment than females (81% versus 89%, respectively; OR=0.52, 95% CI 0.40-0.67, *P*<.001). Data retention rates did not differ by recruitment sources at any follow-up assessment (*P*>.05, data not shown).

### Costs for Participant Recruitment Advertisements

Excluding costs for personnel, the total cost (US $) of recruitment was approximately $84,083.59, or $31.89 per randomized participant. These costs included $1,995.00 for press releases, $4,054.00 for costs associated with mailed letters (ie, postage, printing, mailing supplies, graphic design), $49,791.49 for Facebook ads, $3,506.00 for Google ads, and $7,644.00 for services provided by SSI. The cost per randomized participant for each recruitment source from highest to lowest was $40.51 for Facebook, $34.71 for Google, $20.30 for traditional sources, and $13.95 for the survey panel.

### Rates of Participant Recruitment

Collapsing across all recruitment sources, we enrolled an average of 146 participants per month for the 18-month recruitment period. Recruitment sources varied in rates of participant recruitment (see Table 3).

### Outcome Data Retention

Outcome data retention rates for all assessment points and modalities can be found in Table 4. Overall, data retention rates were 88.85%, 89.16%, and 88.17% for the 3-, 6-, and 12-month follow-ups, respectively.

Collapsing across follow-up assessments and assessment modality, a total of 6995 follow-up assessments were completed. The majority of surveys (6386/6995, 91.29%) were completed online. Of the surveys completed online, most (4261/6386, 66.91%) were completed within 24 hours of an email, earning the US $10 bonus incentive. An additional 894 (14.04%) of online surveys were completed prior to any phone calls; the remaining 1231 (19.33%) were completed after phone calls that began on day 10 of the follow-up period. Of the surveys not completed online, (160/609, 26.27%) were completed by phone, 289 (47.45%) were completed via mailed paper versions, and 160 (26.27%) by postcard. In other words, paper surveys accounted for 289/6995 (4.13%) of all survey responses and the phone and postcard surveys each accounted for 160 (2.28%).

**Table 2:** Baseline predictors of 12-month data retention.

Parameters		Baseline (n)	Not retained (n=327), n (%)	Retained (n=2301), n (%)	Odds ratio (95% CI)	*P* value^a^
**Age (years)**						.18
	18-24	145	24 (16.55)	121 (83.45)	Reference group	
	24-44	984	121 (12.30)	863 (87.70)	1.41 (0.87-2.27)	
	45-64	1312	151 (11.51)	1161 (88.49)	1.52 (0.93-2.39)	
	>65	187	31 (16.58)	156 (83.42)	1.00 (0.55-1.79)	
**Gender**						<.001
	Male	545	103 (18.90)	442 (81.10)	0.52 (0.40-0.67)	
	Female	2083	224 (10.75)	1859 (89.25)	Reference group	
**Race**						.06
	Caucasian	1908	255 (13.36)	1653 (86.64)	Reference group	
	Black/African American	278	21 (7.55)	257 (92.45)	1.88 (1.18-3.00)	
	Other^b^	442	51 (11.54)	391 (88.46)	1.18 (0.86-1.62)	
**Ethnicity**						.93
	Hispanic	222	27 (12.16)	195 (87.84)	1.02 (0.67-1.55)	
	Non-Hispanic	2406	300 (12.47)	2106 (87.53)	Reference group	
**Education**						.14
	≥High school	733	108 (14.73)	625 (85.27)	Reference group	
	Some college or junior college	1363	161 (11.81)	1202 (88.19)	1.29 (0.99-1.67)	
	≥Bachelor’s degree	532	58 (10.90)	474 (89.10)	1.42 (1.01-1.99)	
**Sexual orientation**						.12
	Heterosexual	2375	286 (12.04)	2089 (87.96)	1.42 (1.00-2.04)	
	LGB^c^	253	41 (16.21)	212 (83.79)	Reference group	
**Mental health**						.07
	Screened positive for MHC^d^	1930	251 (13.01)	1679 (86.99)	1.43 (1.05-1.95)	
	Did not screen positive	574	54 (9.41)	520 (90.59)	Reference group	
**Income (US $)**						.52
	≤20,000	735	85 (11.56)	650 (88.44)	0.89 (0.69-1.16)	
	>20,000	1892	242 (12.79)	1650 (87.21)	Reference group	
**Location**						.85
	Metropolitan or micropolitan	2635	293 (12.39)	2072 (87.61)	0.94 (0.64-1.39)	
	Small town or rural	246	32 (13.01)	214 (86.99)	Reference group	

^a^Adjusted for false discovery rate.

^b^Asian, Native American, Native Hawaiian, more than one race.

^c^LGB: lesbian, gay, or bisexual.

^d^MHC: mental health condition.

**Table 3:** Rates of recruitment by source.

Parameter	Recruitment source				
	Traditional (n=298)	Free internet (n=382)	Facebook ad (n=1299)	Google ad (n=101)	Survey panel (n=548)
Duration of use (months)	18 months	18 months	14 months	2 months	5 months
Recruitment rate (persons per month)	16.56	21.22	92.79	50.50	109.60

**Table 4:** Outcome retention rates at 3-, 6-, and 12-month follow-ups by survey modality.

Parameter		Follow-up assessment^a^		
		3-month, n (%)	6-month, n (%)	12-month, n (%)
**Online**				
	≤24 hours of an email	1290 (49.09)	1460 (55.56)	1511 (57.50)
	Before any calls	290 (11.0)	308 (11.72)	296 (11.26)
	After 1-2 calls	292 (11.04)	243 (9.25)	176 (6.70)
	After ≥3 calls	237 (9.02)	142 (5.40)	141 (5.37)
	Online total	2109 (80.25)	2153 (81.93)	2124 (80.82)
**Phone**				
	Within 1-2 calls	34 (1.29)	25 (0.95)	36 (1.37)
	After ≥3 calls	28 (1.07)	14 (0.53)	23 (0.88)
	Phone total	62 (2.36)	39 (1.48)	59 (2.25)
Paper		111 (4.22)	95 (3.61)	83 (3.16)
Postcard		53 (2.02)	56 (2.13)	51 (1.94)
Total number of surveys completed		2335 (88.85)	2343 (89.16)	2317 (88.17)

^a^Data include all randomized participants except for nine that we were unable to classify into one of the five recruitment strategies.

## Discussion

### Study Objectives

Despite the growing popularity of remotely conducted eHealth trials and difficulties regarding participant recruitment and data retention, most studies do not provide detailed accounts or implications regarding such methodologies. As a result, researchers are left with little guidance when planning these methods for eHealth trials. We sought to add to the literature by explicating the recruitment and retention methods used in the WebQuit trial and discussing implications for future online eHealth intervention trials.

### Recruitment and Implications

By implementing a flexible, multi-modal strategy, we recruited and randomized 2637 geographically and demographically diverse adult smokers across the US into a Web-based smoking cessation trial in 18 months. This strategy enabled us to: (1) reallocate resources to methods that were most effective (eg, discontinuing Google ads when Facebook ads were performing better), (2) choose which advertisements to use by monitoring advertisement performance (eg, comparing response rates to Facebook ads), and (3) implement alternative strategies as needed (eg, using an online survey panel to boost recruitment of racial/ethnic minorities).

Unlike findings from our pilot trial [23], participant characteristics varied across recruitment sources. While some differences were intended (eg, a higher proportion of racial and ethnic minority smokers from the online survey panel), other differences were not expected (eg, a more significant proportion of sexual minority smokers from free internet sources; a greater proportion of Black and African American smokers from Google ads). Other studies (eHealth and otherwise) have also found sources of recruitment to be differentially associated with demographic characteristics [22,34,35,44-46]. However, not all studies found the same differences, which may be attributable to differences in recruitment sources used, target population, type of research, and more granular details of recruitment methods (eg, images and words used in advertisements). The variability in participants’ demographic characteristics by recruitment source has implications worth considering for future trials and suggests that restricting recruitment to a single recruitment source may limit the sample diversity and, therefore, the generalizability of trial findings.

A further examination of the recruitment methods lends useful insights for future eHealth trials. For example, although previous studies, including the WebQuit pilot [23], have successfully recruited participants with Google ads [30,47,48], relative to Facebook ads with the same text, Google ads significantly underperformed in terms of the number of participants recruited into the present study, leading to our decision to stop implementing these ads early on. Others have also reported poorer performance of Google relative to Facebook ads [49]. Interestingly, however, Google ads recruited the highest proportions of smokers with lower education, income, and higher proportions of smokers who identified as Black or African American, even though only minimal targeting parameters were used to display some ads in areas based on smoking prevalence, economic status, and rural areas. This suggests that while non-targeted Facebook ads may recruit more substantial numbers of people in shorter amounts of time, Google ads may be more effective at recruiting specific subgroups of individuals with only minimal targeting. Future studies should compare these methods systematically to determine if unique targeting strategies can be used to make the platforms equally effective regarding the rate of recruitment and participant demographics.

Nearly half of the participants in this study were recruited from Facebook. Participants recruited from Facebook were predominantly Caucasian (1116/1299, 85.91%), and female (1101, 84.76%)—characteristics also found in other studies of Facebook-recruited participants [10,12,50]. However, relative to our other sources, Facebook recruited the lowest proportion of young adults aged 18-24 years (24, 1.85%), the highest proportion of older participants aged 45-64 years (902, 69.43%) those aged greater than 65 years (144, 11.09%), and the largest proportion (155, 11.93%) of participants living in small towns or rural areas. Contrary to previous reports that Facebook (and other social media platforms) tends to recruit younger samples [11,50,51], these findings add further support [10,52] that Facebook can recruit older participants, even without targeting for specific age groups. In the context of smoking cessation research, this suggests that, without targeting younger age groups, Facebook ads may be more effective for recruiting an older demographic of smokers wanting to quit. It may also be that Facebook’s algorithm for optimizing ad performance detected that ads worked best among people aged 45-64, then displayed ads to this group more frequently. These findings also suggest that, even without unique targeting parameters, Facebook may effectively reach some hard-to-reach populations, such as the 19% of the US population living in rural areas [53]. Although not done for this study, research suggests that further targeting and adjusting Facebook ad campaigns may increase the likelihood of reaching the desired population [49,54]. For example, different targeting parameters, advertisement images, and wording may be used strategically to recruit highly-specific subsets of participants [27].

Our original sources of recruitment were not recruiting enough racial/ethnic minority smokers, prompting decisions to use an additional source to recruit minority participants (ie, online survey panel) and program our recruitment website to limit enrollment of non-minority smokers. By implementing this combination of strategies, we met our goal of recruiting at least 25% racial/ethnic minority smokers. A closer look at the other characteristics of participants recruited by the survey panel indicates that they tended to be younger and more likely to have an income higher than US $20,000 relative to participants from other sources. This may be due to the nature of individuals who participate in online survey panels. However, since the only specifications we provided SSI were smokers who identified as a racial/ethnic minority, it is possible that providing additional specifications regarding whom we were seeking to recruit would have produced a different sample.

Most participants in this trial were women. Although some studies have successfully recruited samples comprised entirely of males for eHealth research through targeted advertisement campaigns [27,49,54], it is quite common among eHealth intervention trials to have a greater proportion of women compared to men [3,9,24,25,30,55-58]. This is not surprising as women are more likely to utilize eHealth programs [59]. Future studies, particularly those in which specific demographic variables (eg, gender) are deemed essential, should carefully monitor enrollment of participants with selected demographics to help ensure the desired sample is recruited. Much as we limited enrollment of Caucasian participants in this study, future studies might consider limiting enrollment of participants with certain demographics and creating targeted advertising campaigns to recruit participants meeting specific demographic criteria (eg, males). In making decisions regarding which demographic variables to monitor closely and put enrollment limitations on, we encourage researchers to determine who their target population is because a sample’s representativeness is dependent on the characteristics of the target population. For example, characteristics of a representative sample would be different for each of the following target populations: the US adult population, the population of adult smokers, the population of adult smokers who are interested in quitting, and the population of adult smokers interested in quitting with an eHealth intervention.

Overall, when selecting methods for recruitment, researchers should consider many factors including target population, cost, level of reach, targeting abilities, level of ongoing effort required, possible rate of recruitment, and demographic characteristics likely to be recruited from a particular source. For example, in the current study, although the cost per participant enrolled from traditional and free internet sources was relatively low, these methods had limited reach and targeting ability and relatively slow rates of recruitment. In contrast, advertisements through Facebook and Google had a much broader reach, greater ability to target certain populations, faster rates of recruitment, and required low levels of ongoing effort after an initial learning curve. In summary, we highly recommend not only implementing a multi-modal recruitment plan to increase sample diversity, but also monitoring enrollment of participants with characteristics deemed essential for the research question(s). Such practices will not only help ensure that the desired sample is recruited, but can also help researchers determine when alternative recruitment strategies should be implemented to obtain the desired sample.

### Retention Strategy and Implications

Yielding an 88.17% 12-month outcome data retention rate, our sequential, multi-modal participant retention strategy was highly effective at obtaining participant follow-up data. We believe 2 key factors contributed to the high rates of data retention. The first is offering bonus incentives for participants who complete their survey early on. Offering a US $10 bonus to the base incentive resulted in 60.91% (4,261/6,995) of all surveys being completed within 24 hours of receiving 1 of the 3 emails for the Web-based survey. The second is sequentially offering different survey modalities [42], thereby offering multiple opportunities and alternative ways of completing the follow-up surveys. Although very few participants completed the surveys via phone, phone calls appear to have prompted many participants to complete their survey online. Interestingly, of the surveys not completed online, more were completed by mail (the third modality offered) than by phone (the second modality). Future research should empirically examine the possibility of reducing participant- and personnel-related costs associated with these methods (eg, reducing the number of phone calls; sending paper versions of the surveys earlier), and still achieve the high rates of data retention found in this study.

Data retention was not related to recruitment source or participant characteristics measured, with the exception that men were less likely to provide outcome data than women. This gender difference in outcome data retention is consistent with some previous eHealth studies [26,60], though results are mixed [3,57,61]. Reasons for this discrepancy are unclear and are beyond the scope of the present study. Despite this difference, 81.10% of men were retained in the present study at 12-months, which is still quite high for remotely conducted eHealth intervention trials. Future research is needed to understand under what conditions men might be less likely to complete follow-up assessments in eHealth intervention trials and to empirically test strategies to improve their retention rates under those conditions.

### Limitations

The key limitation of these findings is that the study was not designed to compare the effectiveness or cost-effectiveness of the recruitment or retention strategies. Thus, the findings here are meant to describe the methods we used to recruit and retain adult smokers in the WebQuit trial [29], as well as discuss possible methodological implications for future studies. The field would greatly benefit from empirical research designed to test the efficacy and necessity of different recruitment and retention strategies for remotely conducted eHealth trials as well as from more detailed reports regarding recruitment and retention methods to provide generalizable knowledge. Second, online recruitment methods are rapidly evolving. Thus, it is essential to keep in mind that recruitment for this study occurred from 2014-2015. Higher demand for and advancements in technology-based advertising campaigns may limit the generalizability of these findings in the market today. Differences in recruitment data from the WebQuit pilot study conducted in 2010 [23,30] as compared to the present study help emphasize this point. For example, in contrast to the present study, Google ads outperformed Facebook ads in our pilot study. This change may, in part, be due to updates in Facebook advertising options and their proprietary targeting algorithm between the two studies, which have been updated in many ways since this study was completed and can change without notice. Relatedly, our findings regarding Facebook recruitment may not generalize to future trials as user demographics and use trends evolve. Third, because we recruited adult smokers ready to quit smoking, the findings presented here may not generalize to other eHealth trials seeking to recruit other populations. Fourth, although we were able to validate participants recruited from the online panel, all other reports of recruitment source were self-reported, which is subject to recall bias. Finally, as discussed above, despite being diverse in many other ways, our sample was predominately female, which is consistent with a pattern in eHealth trials overall. While our sample may be representative of the population of smokers interested in quitting with an eHealth intervention, it may not be representative of the entire population of smokers.

### Conclusions

Continuous monitoring and refinement of multiple recruitment methods, particularly of online advertising campaigns, was key to our success in recruiting a large, diverse sample of smokers from across the US. Relatedly, offering multiple follow-up survey modalities in sequential order along with time-dependent bonus incentives enabled us to retain most enrolled participants for the duration of the 12-month protocol. Our findings suggest that recruitment sources are associated with demographic differences among participants, but not with differential rates of outcome data retention. Based on the overall success of our participant recruitment and data retention efforts, our experience may serve as an example to others interested in conducting randomized, online clinical intervention trials.

## Abbreviations

DF/HCC: Dana-Farber/Harvard Cancer Center Health Communication Core
LGB: lesbian, gay, or bisexual
MHC: mental health condition
RUCA: Rural-Urban Commuting Area
SSI: Survey Sampling International
